# Supplementary material for: Functional network collapse in neurodegenerative disease
Source: Nat Commun. 2025 Nov 21;16:10273. doi: 10.1038/s41467-025-65156-6 (PMC12639071; doi:10.1038/s41467-025-65156-6)
Supplement: Supplementary file 1 — Supplementary Information [file 41467_2025_65156_MOESM1_ESM.pdf]

Supplementary Information

Supplementary Figures

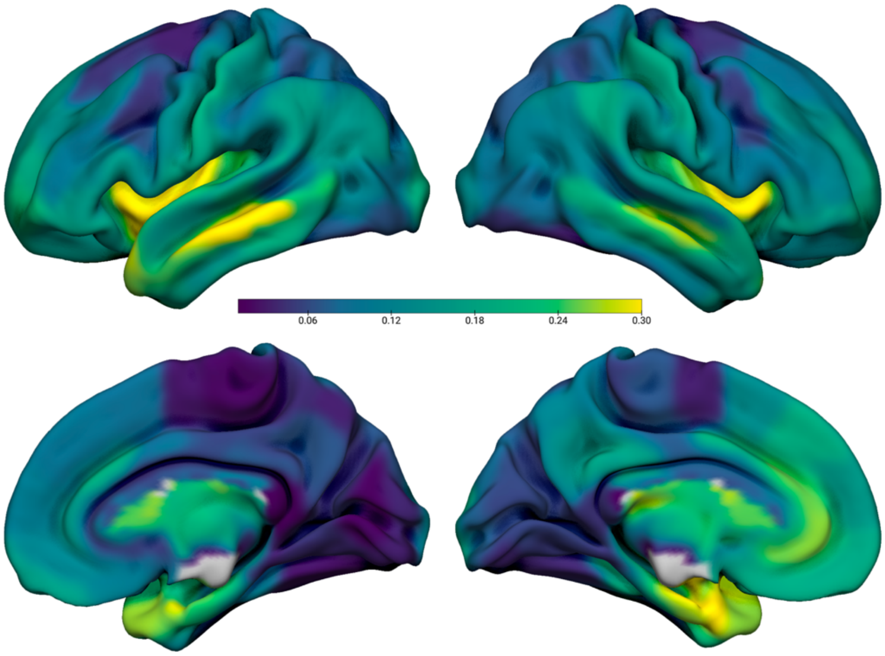

Supplementary Figure 1.
Common regional atrophy pattern across syndromes.
The brain surface map shows the percentage of the 221 patients with significant atrophy (W-score > 1.5) in each of the 246 cortical and subcortical regions.

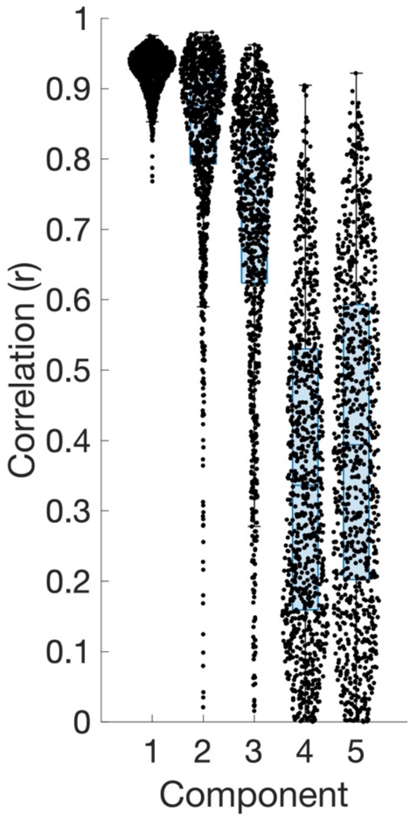

Supplementary Figure 2.
Structural atrophy component reliability.
The correlation of the five structural atrophy component spatial patterns from partial least squares using split-half analysis. The median correlations were S1: r=0.93±0.03, S2: r=0.88±0.14, S3: r=0.77±0.20, S4: r=0.34±0.23, S5: r=0.39±0.24.

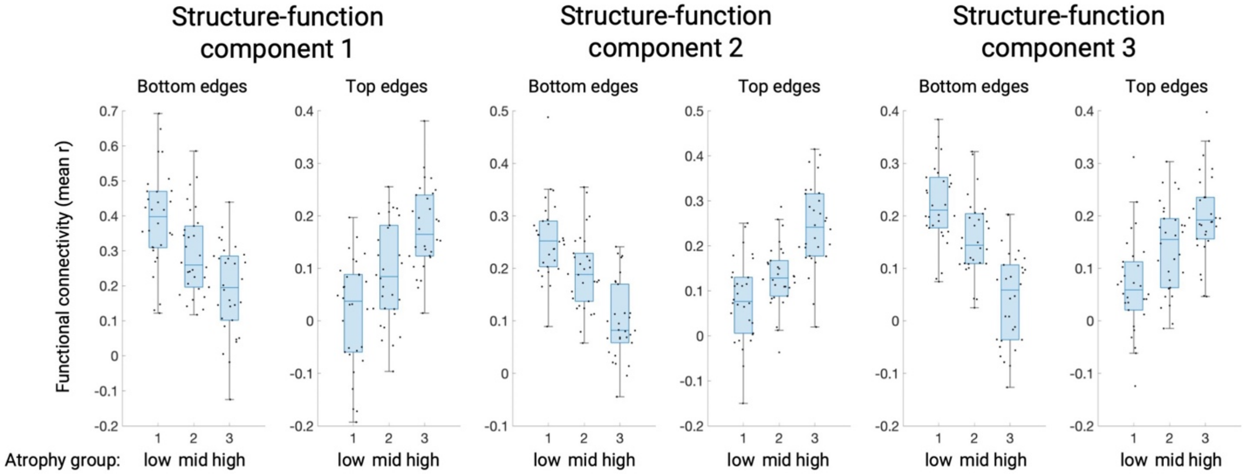

Supplementary Figure 3.
Functional connectivity levels for each structure-function component.

FC edge weights for the bottom/top 1% of edges on each function component. Mean FC edge weights are shown for each component for groups of 30 subjects with the lowest/intermediate/highest atrophy scores. All boxplots show the median, lower and upper quartile range, and the non-outlier minimum/maximum.

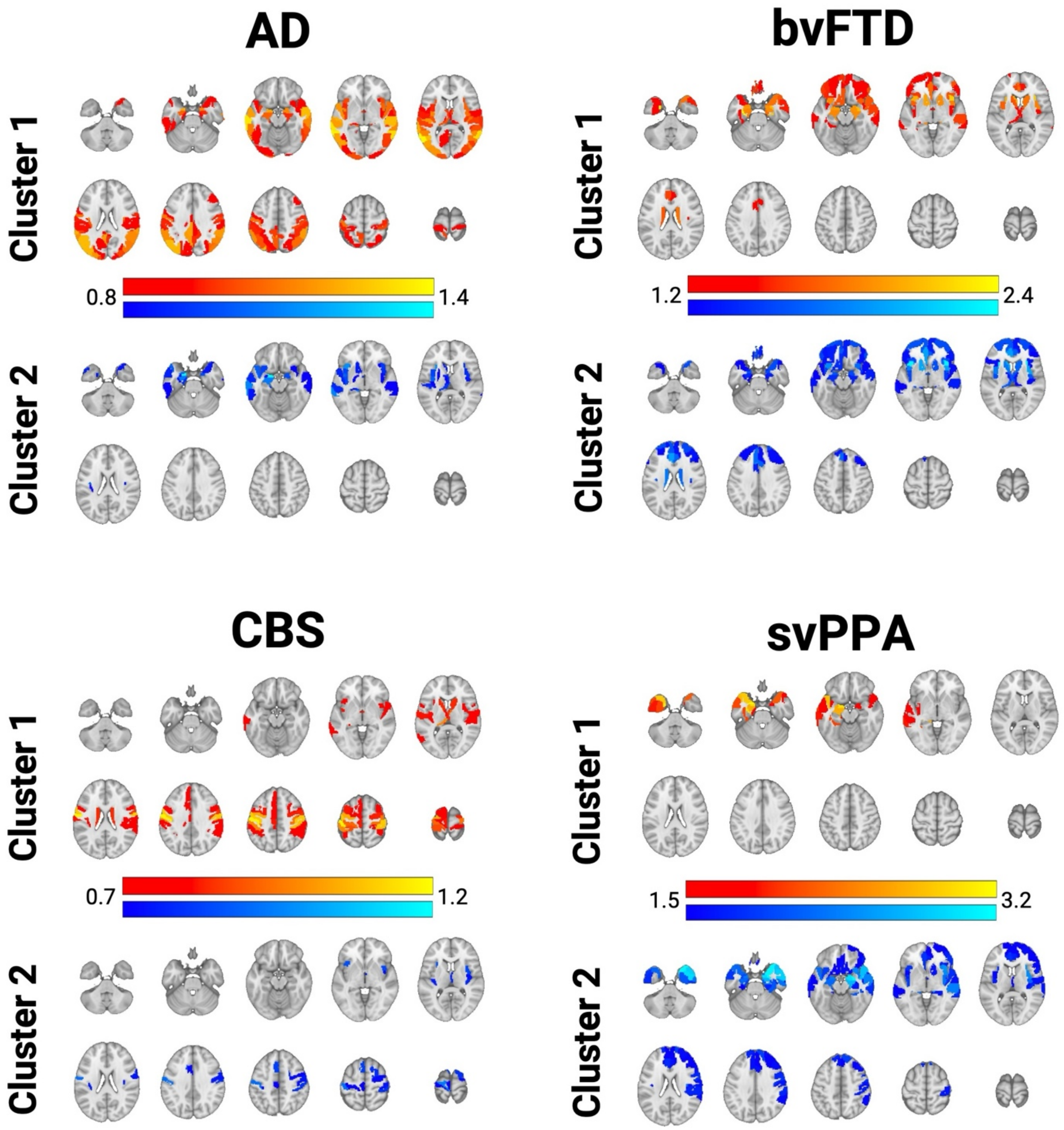

Supplementary Figure 4.
Syndrome atrophy subtypes.

Mean atrophy maps for atrophy subtypes 1 and 2 for AD (n=21/61), bvFTD (n=34/7), CBS (n=10/17), and svPPA (n=33/4). Atrophy values are mean W-scores.

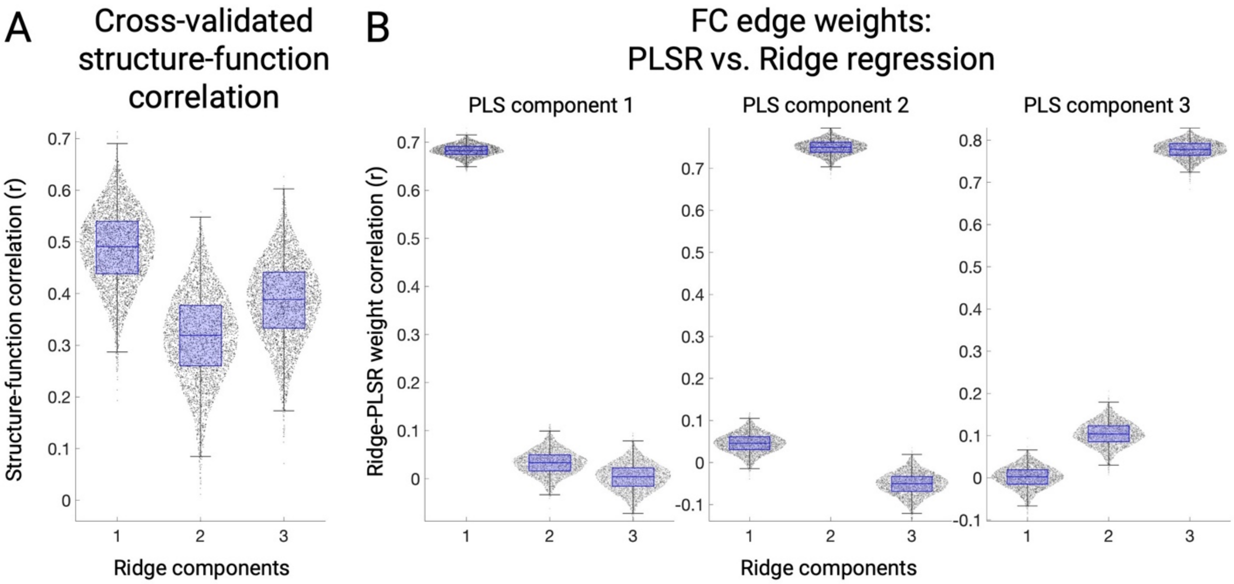

Supplementary Figure 5.
Structure-function component reliability.
A. Out-of-sample correlation coefficients between atrophy component scores and ridge regression-derived functional connectivity scores. Each dot represents a single fold out of four folds per 1000 trials. **B.** Correlation coefficients between ridge regression functional connectivity edge weights (a [30135 x 1] vector) derived separately for each cross-validation fold versus partial least square regression-derived weights. The median correlations between corresponding ridge regression and PLSR components were 1-1: r=0.68, 2-2: r=0.75, 3-3: r=0.78.

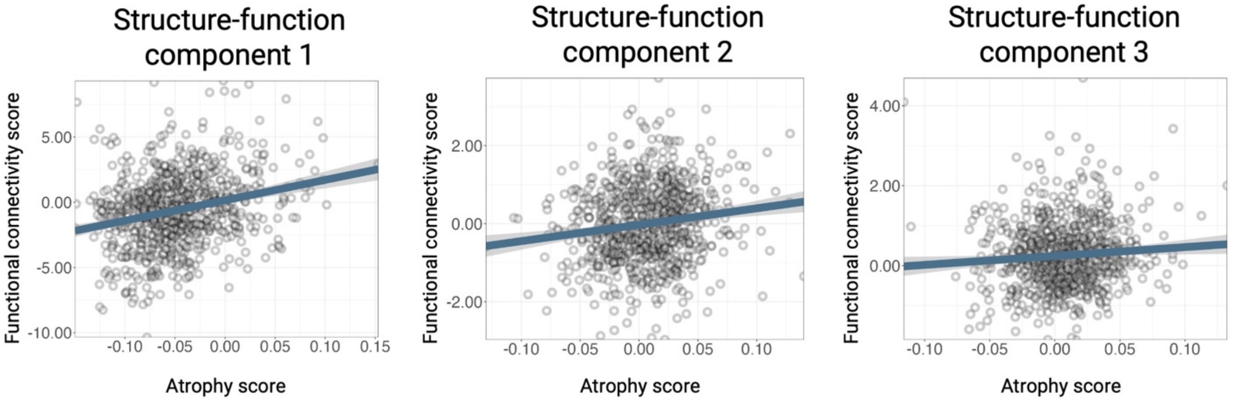

Supplementary Figure 6.
Structure-function component reproducibility.

Structure-function component score correlations for the ADNI replication dataset. The correlation coefficients were SF1: $r=0.25$, $p < 0.001$, SF2: $r=0.15$, $p < 0.001$, SF3: $r=0.08$, $p=0.015$.

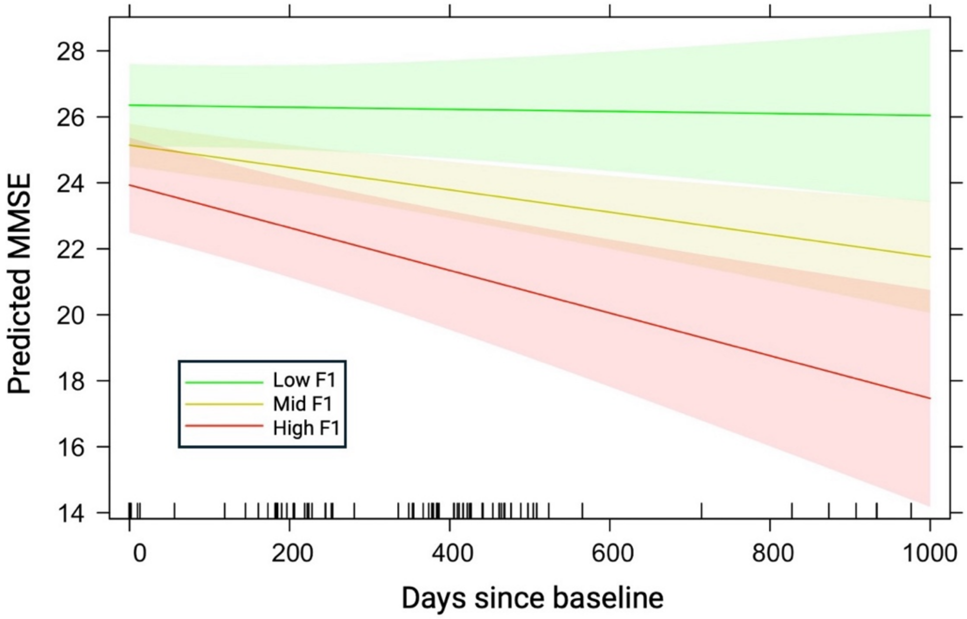

Supplementary Figure 7.
High baseline F1 score predicts accelerated cognitive decline.
The linear effect of baseline functional component 1 (F1) score on longitudinal MMSE in a linear mixed effects model estimating MMSE based on baseline S1, baseline F1, and time (days since baseline).

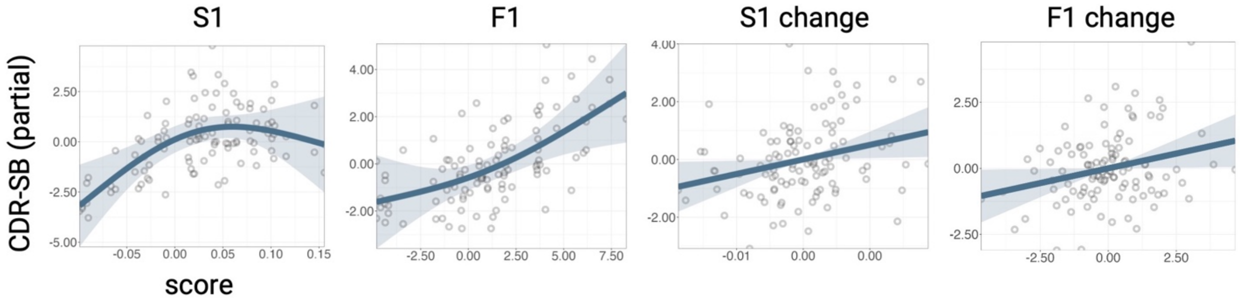

Supplementary Figure 8.
Longitudinal structure-function-clinical relationships.
Longitudinal relationship effect plots showing the partial relationship of CDR-SB with structure component 1 (S1) mean (between-subject), function component 1 (F1) mean (between-subject), S1 change (within-subject), and F1 change (within-subject).

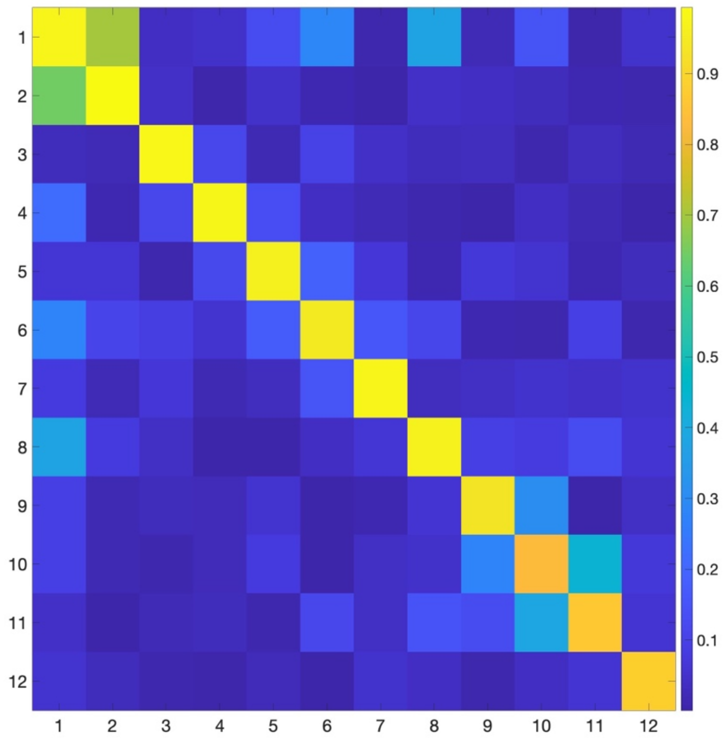

Supplementary Figure 9.
Functional gradient reproducibility.
Spatial correlations between the fMRI PCA spatial components (gradients; n=246 regions per component) derived from the independent cognitively normal cohort (n=321, rows) and the main combined patient and control cohort (n=321, columns).

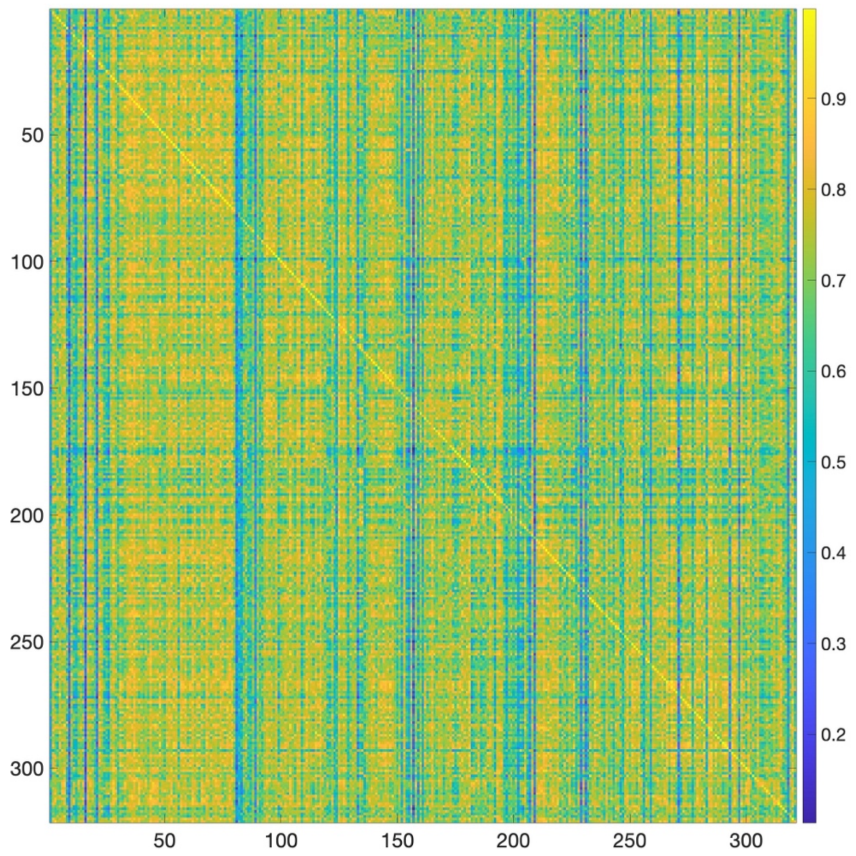

Supplementary Figure 10.
Correspondence of real and simulated functional connectivity.
Correlations between each subject's actual [246 x 246] FC matrix (rows) and simulated FC matrix based on their individual coupled oscillator model (columns).

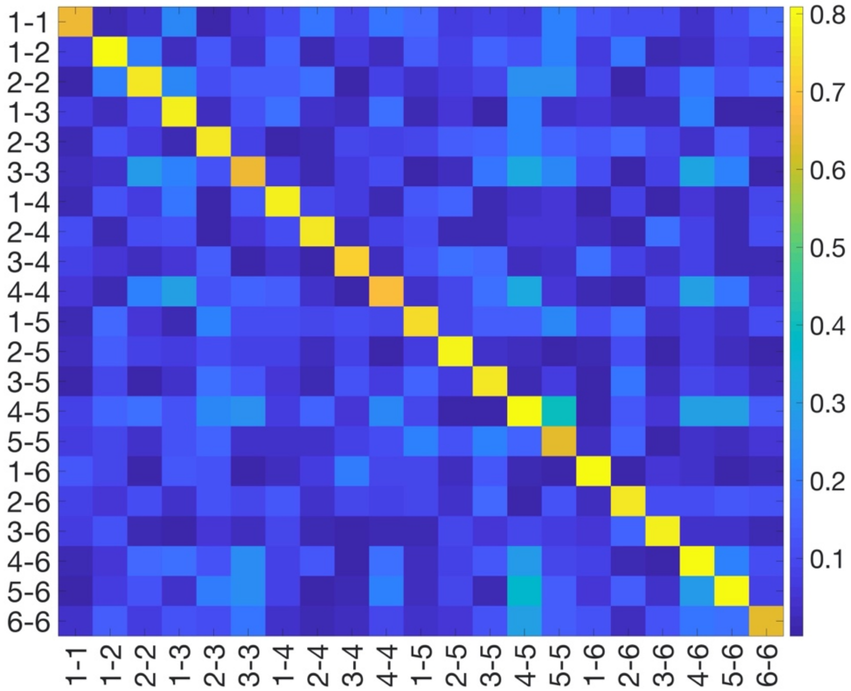

Supplementary Figure 11.
Correspondence between gradient statistical and dynamical properties.
Correlations between observed gradient variance/covariance and coupling parameter-derived gradient amplitude/angles.

Supplementary Results.

Diagnosis	Mean atrophy (W)	S1	S2	S3	Mean FC	F1	F2	F3	Mean FD
**AD**	0.70 ± 0.43	-0.02 ± 0.05	-0.03 ± 0.04	-0.04 ± 0.04	0.15 ± 0.05	70.94 ± 283.53	-87.42 ± 272.96	-104.02 ± 142.42	0.23 ± 0.11
**bvFTD**	0.94 ± 0.43	-0.05 ± 0.05	0.02 ± 0.05	0.06 ± 0.06	0.15 ± 0.05	197.38 ± 187.53	47.56 ± 245.96	133.12 ± 157.27	0.28 ± 0.13
**CBS**	0.43 ± 0.48	0.01 ± 0.05	-0.04 ± 0.04	0.02 ± 0.05	0.13 ± 0.04	74.40 ± 324.68	-157.45 ± 235.50	61.30 ± 166.65	0.31 ± 0.13
**nfvPPA**	0.61 ± 0.39	-0.01 ± 0.04	-0.02 ± 0.05	0.03 ± 0.06	0.15 ± 0.04	90.85 ± 305.88	-44.86 ± 225.52	85.95 ± 165.70	0.27 ± 0.14
**svPPA**	0.66 ± 0.44	-0.03 ± 0.05	0.09 ± 0.05	-0.05 ± 0.05	0.16 ± 0.05	144.43 ± 346.94	190.24 ± 278.96	-98.84 ± 132.55	0.25 ± 0.12
**CN**	0.04 ± 0.30	0.05 ± 0.03	-0.01 ± 0.03	0.01 ± 0.02	0.17 ± 0.05	-243.51 ± 340.38	39.56 ± 227.69	21.52 ± 123.48	0.26 ± 0.12

Supplementary Table 1. Mean scores for each syndrome for structural atrophy W-score, Structure components 1-3 (S1/S2/S3), mean functional connectivity (FC), Function components 1-3 (F1/F2/F3), and fMRI framewise displacement (FD).

Assessing stage and subtype within each syndrome

Across syndromes, between-subject dispersion in global atrophy exceeded controls (all FDR-corrected q < 0.05), indicating greater within-syndrome heterogeneity. Within each syndrome, mean atrophy was strongly predicted by three structural

components ($R^2 > 0.8$; FDR-corrected $q < 0.05$), consistent with a continuous severity spectrum. Conditioning on this severity axis, model comparison favored two subtypes over one in AD (ΔBIC=68.1, 21/61 subjects in cluster 1/2), with more widespread cortical versus limbic-focused atrophy; bvFTD (ΔBIC=4.0, 34/7 subjects in cluster 1/2), with more ventral versus dorsal frontal atrophy; CBS (ΔBIC=14.7, 10/17 subjects in cluster 1/2), with more widespread versus focal sensorimotor atrophy; and svPPA (ΔBIC=39.4, 33/4 subjects in cluster 1/2), with more left-lateralized versus right-lateralized anterior temporal atrophy; but not nfvPPA (ΔBIC=−4.3), indicating discrete variation beyond severity in four syndromes.

Supplementary Results for Supplementary Figure 2.

1000 trials of independent PLSR were run on the first and second halves of the subjects, randomly split with balanced syndrome classes for each trial. The structure component loading vectors were correlated between split halves for the 1000 trials for the first five PLSR components. The median correlations were S1: r=0.93±0.03, S2: r=0.88±0.14, S3: r=0.77±0.20, S4: r=0.34±0.23, S5: r=0.39±0.24. The most substantial drop in reliability was between components 3 and 4.

Supplementary Results for Supplementary Figure 3.

For the first three structure-function components, subjects were sorted based on their structural score for that component and binned into groups of 30. For a given component, FC edges were sorted based on their PLSR weight. The bottom/top 1% of edges (300 edges) were kept and the mean FC weight for these two sets were computed for each subject. These mean edge weights were statistically compared for the groups of 30 subjects with low/middle/high atrophy on that component, both for the bottom and top FC edges. The edge weights were always statistically significant between low and high subjects (all $p < 0.001$, see table) and were significant at a less stringent threshold ($p < 0.01$) in all other tests. This indicated that the partial FC variance captured by the FC scores was substantial enough to capture significant differences in overall FC edge weights between groups of subjects.

	low vs. mid, t	low vs. mid, p	mid vs. high, t	mid vs. high, p	low vs. high, t	low vs. high, p
SF1, bottom	2.86	0.005	3.22	0.002	5.77	< 0.001
SF1, top	-2.89	0.005	-3.74	< 0.001	-6.69	< 0.001
SF2, bottom	3.24	0.002	4.95	< 0.001	8.11	< 0.001
SF2, top	-2.51	0.01	-5.20	< 0.001	-6.87	< 0.001
SF3, bottom	3.32	0.002	5.52	< 0.001	8.44	< 0.001

SF3, top	-3.19	0.002	-2.80	0.006	-6.01	< 0.001

Structure-function component validation

We confirmed the structure-function relationship reliability in two ways. First, we used ridge regression with four-fold cross-validation (**Supplementary Methods**). We found that the three primary components each had significant structure-function correlation in the left-out fold (SF1: median r=0.49, median FDR $p < 0.05$; SF2: r=0.32, p=0.003; SF3: r=0.39, FDR $p < 0.05$; **Supplementary Figure 5A**). Importantly, the FC edge weights associated with each atrophy component in cross-validated subsamples strongly matched the FC edge weights for the corresponding component from the full sample (**Supplementary Figure 5B**). Second, we assessed these structure-function components in a separate replication dataset from the Alzheimer's Disease Neuroimaging Initiative (ADNI3) sample, comprised of 421 cognitively normal subjects and 56 subjects with Alzheimer's disease from 35 scanning sites. Structure and function scores for each component were significantly correlated (SF1: r=0.25, t=4.94, FDR $p < 0.05$; SF2: r=0.15, t=3.81, FDR $p < 0.05$; SF3: r=0.08, t=2.42, p=0.015; **Supplementary Figure 6**).

Structure-function-cognition reliability

First, we tested if baseline S1 and F1 scores could predict future cognitive change in subjects with longitudinal cognitive data. The significant predictors of more rapid longitudinal MMSE decline over three years were higher baseline F1 (t=-1.98, uncorrected p= 0.04), time x baseline F1 (t=-2.72, FDR $p < 0.05$; **Supplementary Figure 7**; higher baseline F1: more rapid MMSE decline), and time x baseline S1 x baseline F1 (t=-2.06, uncorrected p= 0.04; higher baseline S1 and F1: trend for more rapid MMSE decline). Thus, individuals with worse F1 scores had more rapid cognitive decline. Second, we estimated CDR-SB scores in the ADNI replication dataset using 774 scans with associated cognitive test scores. This model explained 33.2% of the CDR-SB variance with the strongest predictions from S1 (F=57.99, FDR $p < 0.05$), F1 (F=12.13, FDR $p < 0.05$), F3 (F=21.33, FDR $p < 0.05$), and S2 (F=16.63, FDR $p < 0.05$). This indicated that the same structure-function patterns were present in different patients with dementia and had a largely similar impact on cognitive impairment. Third, we evaluated the longitudinal relationship between S1, F1, and CDR-SB using baseline and follow-up data from a subset of 47 patients and 6 cognitively normal subjects from the main dataset (mean visit interval=1.1±0.5 years, range=0.4-2.6 years). In this smaller longitudinal model, we found a statistical trend for within-subject CDR-SB change correlating with F1 change (t=2.05, p=0.04; **Supplementary Figure 8**) and S1 change (t=2.15, p=0.04). Between-subject CDR-SB mean had a trend relationship to S1 mean (F=6.06, p=0.02) and F1 mean (F=3.34, p=0.03) as expected from the cross-sectional model. A brain-only model showed that overall F1 significantly correlated with S1 mean (F=21.42, $p < 0.001$) and had a trending correlation with S1 change (F=6.21, p=0.02).

Supplementary Results for Supplementary Figure 9.

The spatial gradient patterns were highly consistent in the independent cognitively normal cohort and the primary cohort. The median spatial correlation was r=0.98 for gradients 1-6 (max=0.99, min=0.95) and r=0.97 for gradients 1-12 (max=0.99, min=0.83).

Supplementary Results for Supplementary Figure 10.

We validated the accuracy of this modeling approach by simulating gradient timeseries based on subject-specific eigenmodes and comparing simulated to actual FC patterns. We found that each subject's simulated and actual FC was significantly more similar than to simulated FC from the other subjects (self-actual vs. self-simulated, median r=0.96; self-actual vs. other-simulated, median r=0.72; t=31.46, FDR $p < 0.05$; **Supplementary Figure 10**).

Supplementary Methods.

Structural image processing

MPRAGE scans were visually assessed by trained technicians and scans with excessive motion artifact (ringing or blurring) were excluded. MPRAGE scans for all time points for a given subject that passed visual inspection were registered using the serial longitudinal registration in SPM12 [1]. Default parameters were used for warping regularization and bias regularization. Jacobian determinant and divergence maps were produced that represent the amount of longitudinal brain contraction and expansion. We then applied unified normalization/segmentation to register the midpoint average T1 images to the MNI152NLin6Asym standard space [2] with light regularization, a 60 mm bias FWHM cutoff, and Gaussians per tissue type of [2,2,2,3,4,2]. The gray matter tissue segmentation for the midpoint average was multiplied by the deformation fields for each time point to obtain time point-specific gray matter maps. These images were then warped to standard space using the deformation fields from the unified normalization/segmentation procedure. The resulting normalized gray matter maps were smoothed with an 8 mm FWHM Gaussian kernel.

We derived voxelwise gray matter tissue probability maps from an independent set of cognitively normal control subjects (n=397) using the same structural image processing methods. These subjects had the following characteristics: mean age=69.3±8.8; 239 female/158 male; 345 right-handed/43 left-handed; 1.5T/3T Trio/3T Prisma/4T=58/144/140/52). We ran multiple regression for each voxel to estimate gray matter volume as a function of age, sex, handedness, total intracranial volume, and MRI scanner identity. For the 321 subjects in the primary analysis, we entered their demographic values into this regression model to estimate their gray matter volume in each voxel. The voxel W-score was calculated as the difference between actual gray matter volume and the estimated gray matter volume, divided by the standard deviation of the model fit in the reference control sample [3]. W-scores were used as the measurement of gray matter atrophy throughout the study.

Functional image processing

Functional MRI scans were processed using fMRIPrep [4] (RRID:SCR_016216). For anatomical image processing, the MPRAGE images were corrected for intensity non-uniformity with N4BiasFieldCorrection in ANTs (Avants et al., 2008) (RRID:SCR_004757), and used as the T1-weighted (T1w) reference throughout the workflow. The T1w reference was skull-stripped with a Nipype [5] (RRID:SCR_002502) implementation of the antsBrainExtraction.sh workflow using OASIS30ANTs as target template. Brain tissue segmentation of cerebrospinal fluid (CSF), white-matter (WM) and gray-matter (GM) was performed on the brain-extracted T1w using FSL fast (https://fsl.fmrib.ox.ac.uk/fsl/fslwiki; RRID:SCR_002823). Volume-based spatial normalization to the MNI152NLin6Asym standard space was performed through nonlinear registration with antsRegistration, using brain-extracted versions of both T1w reference and the T1w template.

For functional image processing, the first five volumes were removed to allow for scanner stabilization. A reference volume and its skull-stripped version were generated

by fMRIPrep. The BOLD reference was then co-registered to the T1w reference using FSL flirt with 6-degrees-of-freedom affine registration. Co-registration was configured with nine degrees of freedom to account for distortions remaining in the BOLD reference. Head-motion parameters with respect to the BOLD reference (transformation matrices, and six corresponding rotation and translation parameters) were estimated using FSL mcflirt and were used to compute the framewise displacement (FD). BOLD runs were slice-time corrected using AFNI 3dTshift (https://afni.nimh.nih.gov/; RRID:SCR_005927). The BOLD images were realigned from native to MNI152NLin6Asym standard space using antsApplyTransforms, configured with Lanczos interpolation, with a single interpolation step by composing transformations for head-motion and co-registrations to anatomical and output spaces. Images were spatially smoothed with a 6mm FWHM (full-width half-maximum) kernel using FSL susan. Confounding CSF and WM timeseries were calculated based on the preprocessed BOLD images, deriving average signals using the subject-specific anatomically derived tissue masks after erosion. The confound timeseries for head motion estimates, CSF, and WM were expanded to include the temporal derivatives and quadratic terms [6]. Bandpass filtering in the frequency range 0.008-0.08 Hz was performed on the confound timeseries and BOLD images using fslmaths and AFNI 3dBandpass respectively. We did not perform global signal regression and instead assessed global signal variance as a disease-relevant variable of interest. The global signal was computed as the mean BOLD signal across the 246 regions (see below) at each timepoint. Confound timeseries were then regressed out of the BOLD images using fslglm. Scans were standardized voxelwise to have mean=0 and standard deviation=1 across time. Subjects with greater than 0.55 mm mean FD were excluded from subsequent analysis [7]. From the pool of all fMRI scans for all available control and patient subjects (n=1591), this resulted in the exclusion of 194 scans (12.2%). We performed a subsequent data-driven denoising procedure using PCA to remove scans with implausible functional connectivity patterns likely due to noise from the scanner hardware (field instabilities) and the subject (head motion, heartbeat, respiration). PCA has previously been applied for outlier detection in fMRI data [8]. Here, all subject's functional connectivity matrices (see below) were flattened and combined into one single matrix ([1591 scans x 30135 edges]). PCA was run on this matrix and subjects with an outlying score on the first component (> 1 standard deviation above the mean) were flagged. This excluded 288 scans and left an available pool of 1108 scans. The excluded scans included all diagnoses (33 AD, 31 bvFTD, 18 CBS, 12 nfvPPA, 41 svPPA, 347 CN) and did not differ from the included scans in terms of mean atrophy (excluded mean region W-score=0.29 vs included mean W-score=0.26). There was a slight trend for excluded scans to have a lower CDR score than the included scans (mean=3.16 vs 2.66, t=1.83, p=0.07).

Brain structure-function statistical analysis

We ran PLSR to derive five components and used split-half analysis to determine which components had sufficient reliability. Here we focused on the structural component reliability and describe our procedure for functional component reliability in

the next section. We generated 1000 random splits of the 321 subjects into two equally sized groups, each time balancing the number of subjects with each clinical syndrome. We ran 1000 trials of independent PLSR on the first and second halves of the subjects. The structure component loadings were compared for each of the 1000 trials using correlation. Analysis revealed that the first three components had acceptable reliability. We obtained the atrophy and FC scores for each subject for these three components and computed the structure-function correlation from these scores to measure the strength of each independent structure-function relationship.

We validated the reliability of the structure-function relationships by performing ridge regression with cross-validation. For this analysis, we could not use atrophy component scores from our PLSR model because these were derived based on their covariance with FC and would contaminate cross-validation. Instead, we derived atrophy component scores using PCA on the atrophy data alone. The spatial patterns of the first three PCA-derived atrophy components were essentially identical to the first three PLSR atrophy components (Component 1, r=0.999; Component 2, r=0.98, Component 3, r=0.97). Ridge regression was run using scikit-learn [9] (https://scikit-learn.org/stable/modules/generated/sklearn.linear_model.Ridge.html). We estimated models of the relationship between each atrophy component (one score per subject per component) and brain-wide FC weights (30135 features per subject). Stratified cross-validation was run to measure model out-of-sample accuracy with four folds with 240 train subjects and 81 test subjects per fold, balanced for the number of subjects with each syndrome. We ran 1000 trials with an empirically selected alpha value of 1000, which produced optimal cross-validation accuracy. Ridge coefficients for each component were averaged across 1000 trials and 4 folds to obtain the average coefficients. Individual subject functional scores for each component were derived by taking each subject's score from their left-out fold for each trial and averaging across the 1000 trials. Deriving these out-of-sample ridge FC scores also served to decorrelate structure and function scores sufficiently to use them as independent predictors of neuropsychological scores.

We evaluated the reliance of the structure-function components on the cross-decomposition algorithm by comparing our default PLSR method to ‘PLSCanonical’ in scikit-learn. PLSCanonical finds common modes shared between X (atrophy) and Y (functional connectivity) in a symmetric, correlation-like way rather than PLSR’s asymmetric, regression-like objective of using X to predict Y. Nonetheless, we found that the first three structure-function components from PLSCanonical corresponded to those from PLSR, with the X and Y components matching in order (S1: r=0.70; S2: r=0.90, S3; r=0.91; F1: r=0.99; F2: r=0.96; F3: r=0.95). Thus the structure-function components appear robust to the choice of the algorithm.

Dynamical systems modeling

Gradient timeseries simulations were run using the real gradient timeseries and first derivatives for each timepoint as the initial condition and running for the number of timepoints in that subject’s scan (235 or 555 for Trio or Prisma). The [235/555 x 6] gradient timeseries were matrix multiplied by the [6 x 246] region gradient weights to

obtain [246 x 235/555] region timeseries, from which [246 x 246] FC matrices were computed. These 235/555 matrices were averaged to produce the subject's simulated FC matrix. For the actual data, the FC matrices were derived from the six gradients' timeseries. Simulated and real FC matrices for each condition were statistically compared using Pearson correlation on the matrix upper triangle edge weights.

We performed an illustrative eigenmode analysis on subjects with the lowest and highest function component 1 (F1) scores, associated with the lowest or highest overall mean atrophy. We sorted subjects based on F1 scores and grouped the five subjects with the lowest or highest scores, limited to only subjects with Siemens Trio scans. Gradient timeseries for the five subjects were concatenated into a [1175 x 6] matrix, from which we computed coupling parameters and derived the eigenmodes. The gradient timeseries were simulated for 1175 timepoints, of which the first 600 timepoints are displayed in **Figure 5**. The region timeseries and FC matrices were computed and compared for both subject groups.

Brain-behavior statistical analysis

In the ADNI validation dataset, a longitudinal model was used to estimate the relationship between S1, F1, and CDR. 53 subjects (47 patients, 6 cognitively normal subjects) had longitudinal imaging and cognitive data (mean visit interval=1.1±0.5 years, range=0.4-2.6 years) that passed all quality control tests. S1 and F1 scores were measured for follow-up scans using the linear weights from atrophy PLSR component S1 for structure and from ridge regression for function. We specified a mixed effects model using 'mgcv' with CDR-SB score from each scan-associated visit (106 total) as the outcome variable. The model estimated both between-subject and within-subject variation as in [10] by including S1 subject mean (averaged across timepoints; non-linear basis k=3 to match the cross-sectional model), S1 longitudinal change (difference from the subject's mean, varying within-subject across timepoints; k=1 to limit model degrees of freedom), F1 mean (k=3), F1 longitudinal change (k=1), age, sex, years of education, and random intercepts for each subject. In a complementary model, F1 was estimated as a function of S1 baseline (k=3), S1 change (k=3), age, sex, years of education, visit interval, and random intercepts for each subject. Due to the unique nature of this model as a replication sub-analysis including longitudinal data, we report trend-level statistics with $p < 0.05$ where the direction of the result matched the direction from the larger discovery model.

Replication analysis

A replication dataset was compiled from subjects in the ADNI3 study [11], obtained from the Alzheimer's Disease Neuroimaging Initiative (ADNI) database (https://adni.loni.usc.edu/). The ADNI was launched in 2003 as a public-private partnership, led by Principal Investigator Michael W. Weiner, MD. The primary goal of ADNI has been to test whether serial magnetic resonance imaging (MRI), positron emission tomography (PET), other biological markers, and clinical and neuropsychological assessment can be combined to measure the progression of mild cognitive impairment (MCI) and early Alzheimer's disease (AD). For up-to-date

information, see www.adni-info.org. We included subjects with a diagnosis of cognitively normal or Alzheimer's disease dementia who received structural MRI and resting state functional MRI scans. A total of 966 visits from 573 subjects (CN n=500, AD n=73) at 68 sites were obtained. We included the subset of scans that were from sites with 10 or more subjects, and that satisfied fMRI motion criteria (mean framewise displacement <= 0.55 mm), resulting in 821 scans. These subjects had the following characteristics: diagnosis, CN n=421, AD n=56; mean age, CN=72.9±8.1 years, AD=75.6±8.1 years; 277 female/200 male; 437 right-handed/40 left-handed; mean interscan interval=1.75±0.7 years). Scans were run on 3T scanners including Siemens (Prisma, n=441; Biograph, n=5; Skyra, n=12; Trio, n=48; Verio, n=91), Philips (Medicare Ingenia, n=54; Achieva, n=78), or GE (Medical Systems Discovery, n=209; Signa n=23). The typical sMRI acquisition parameters were acquisition time: 6:20; sagittal slice orientation; thickness: 1.0 mm; field of view: 208x240x256 mm; isotropic voxel resolution: 1mm^3; TR: 2300 ms; TE: 3 ms; TI: 900 ms. The typical single-band tf-fMRI acquisition parameters were acquisition time: 10:00; axial orientation with interleaved ordering; field of view: 220x220x163 mm; matrix size: 92x92, effective voxel resolution: 2.2x2.2x2.2 mm; TR: 3000 ms, for a total of 560 volumes; TE: 30 ms; with instructions to remain awake with eyes open. The typical multi-band tf-fMRI acquisition parameters were acquisition time: 10:00; axial orientation with interleaved multi-slice mode and multiband acceleration=8; field of view: 220x220x160 mm; matrix size: 92x92, effective voxel resolution: 2.5x2.5x2.5 mm; TR: 670 ms; TE: 30 ms. We included n=189 multiband scans (TR=0.67/0.79ms, only from Siemens Prisma/Prisma Fit/Syra scanners) and n=777 single-band scans (TR=3/3.15s). CDR-SB scores were available for 774/821 scans. Images were processed with the same pipelines used for the main dataset. Atrophy W-maps and [246 x 1] region atrophy vectors were derived for each structural scan using the same W-score model with the default intercept. [246 x 246] FC matrices were obtained for each functional scan. We harmonized atrophy and FC data across sites by running ComBat on the atrophy/FC values for all scans, controlling for patient/control status, age, and sex. We then computed atrophy component scores for components 1-3 using the atrophy PCA loadings. Functional connectivity component scores were computed for components 1-3 using the average ridge regression coefficients. Structure-function relationships were estimated using a mixed effects linear model with functional component score as the response variable, fixed effects for the corresponding structural component score, mean age, sex, years of education, and framewise displacement, and random intercepts for subject and site. CDR scores were estimated using a mixed effects GAM with CDR-SB as the response variable, non-linear fixed effects for S1-S3 and F1-F3 (all with a non-linear basis k=3), linear fixed effects for mean age, sex, years of education, and framewise displacement, and random intercepts for site.

Assessing stage and subtype within each syndrome

We tested for the presence of meaningful atrophy stages and subtypes within each syndrome. We analyzed each syndrome separately (AD, bvFTD, CBS, nfvPPA, svPPA, CN) using the three PLS structure components. We used two complementary

measures of disease stage: mean overall atrophy, a more coarse measure of disease stage; and Euclidean distance to the cognitively normal group mean, a more context-specific measure of disease stage. We first tested whether mean overall atrophy was captured by the three structure components. We fit a linear model within each syndrome: mean_atrophy ~ S1 + S2 + S3 (with intercept), and calculated the model R^2 and p-value. We also tested whether mean overall atrophy variability in that syndrome exceeded controls using a one-sided F-test on variances (syndrome vs CN). We considered stages present when the regression was significant with a non-trivial effect ($p < 0.05$ and $R^2 \geq 0.10$) and the variance exceeded controls ($p < 0.05$).

After establishing a relationship between mean overall atrophy and the structure components, we derived a proxy for disease stage by computing each subject's Euclidean distance from the CN centroid, which we refer to as 'CN distance'. We used this measure to evaluate the possibility of syndrome-specific atrophy subtypes. We removed stage effects within each syndrome by orthogonalizing S1/S2/S3 against CN distance. With these residuals, we tested whether two clusters fit better than one using two metrics: 1) the mean silhouette for k=2 (k-means), and 2) the BIC difference for Gaussian mixtures, ΔBIC = BIC(1-component) − BIC(2-component), using fitgmdist in Matlab. We called atrophy subtypes present when $\Delta BIC > 0$ and mean silhouette ≥ 0.25.

Gradient-to-Eigenmode Workflow

The procedure for taking individual subject fMRI data and deriving gradients, eigenmodes, and functional connectivity (FC) metrics is:

1. Dimensionality reduction. Concatenate region-level fMRI timeseries from an independent cognitively normal cohort and run PCA; retain first 6 components (spatial "gradients").
2. Project patient data. Multiply each patient's region timeseries by the gradient loading matrix to obtain 6 gradient timeseries per subject.
3. Build coupled oscillator model. Calculate each gradients' first and second temporal derivatives, the velocity and acceleration. Estimate second order linear ordinary differential equations: each gradient's acceleration (x'') as a linear function of all gradients' positions (x) and velocities (x') (13 parameters × 6 equations = 6×13 coupling matrix).
4. Eigendecomposition. Compute eigenvectors and eigenvalues of the coupling matrix, which are the eigenmodes; extract gradient specific amplitudes and phase angles. Optionally, simulate gradient timeseries using the coupled oscillator model equations with empirically selected initial conditions for all gradients' positions and velocities.
5. Link to FC. Reconstruct FC matrices from real or simulated gradient timeseries; compare amplitude/phase shifts to empirical hypo/hyper connectivity patterns.

Supplementary References

1. Ashburner, J. & Ridgway, G. R. Symmetric diffeomorphic modeling of longitudinal structural MRI. *Front Neurosci* **6**, 197 (2012).
2. Ashburner, J. & Friston, K. J. Unified segmentation. *Neuroimage* **26**, 839–851 (2005).
3. Joie, R. L. *et al.* Region-Specific Hierarchy between Atrophy, Hypometabolism, and β-Amyloid (Aβ) Load in Alzheimer's Disease Dementia. *J. Neurosci.* **32**, 16265–16273 (2012).
4. Esteban, O. *et al.* fMRIPrep: a robust preprocessing pipeline for functional MRI. *Nat. Methods* **16**, 111–116 (2019).
5. Gorgolewski, K. *et al.* Nipype: a flexible, lightweight and extensible neuroimaging data processing framework in python. *Front Neuroinform* **5**, 13 (2011).
6. Satterthwaite, T. D. *et al.* An improved framework for confound regression and filtering for control of motion artifact in the preprocessing of resting-state functional connectivity data. *NeuroImage* **64**, 240–256 (2013).
7. Parkes, L., Fulcher, B., Yücel, M. & Fornito, A. An evaluation of the efficacy, reliability, and sensitivity of motion correction strategies for resting-state functional MRI. *NeuroImage* **171**, 415–436 (2018).
8. Mejia, A. F., Nebel, M. B., Eloyan, A., Caffo, B. & Lindquist, M. A. PCA leverage: outlier detection for high-dimensional functional magnetic resonance imaging data. *Biostatistics* **18**, 521–536 (2017).
9. Pedregosa, F. *et al.* Scikit-learn: Machine Learning in Python. *J. Mach. Learn. Res.* **12**, 2825–2830 (2011).
10. Staffaroni, A. M. *et al.* The Longitudinal Trajectory of Default Mode Network Connectivity in Healthy Older Adults Varies As a Function of Age and Is Associated with Changes in Episodic Memory and Processing Speed. *J. Neurosci.* **38**, 2809–2817 (2018).
11. Weiner, M. W. *et al.* The Alzheimer's Disease Neuroimaging Initiative 3: Continued innovation for clinical trial improvement. *Alzheimers Dement* **13**, 561–571 (2017).
