# Supplementary material for: Functional network collapse in neurodegenerative disease
Source: Nat Commun. 2025 Nov 21;16:10273. doi: 10.1038/s41467-025-65156-6 (PMC12639071; doi:10.1038/s41467-025-65156-6)
Supplement: Supplementary file 2 — Reporting Summary [file 41467_2025_65156_MOESM2_ESM.pdf]

nature portfolio

Corresponding author(s): Jesse Brown

Last updated by author(s): Apr 16, 2025

Reporting Summary

Nature Portfolio wishes to improve the reproducibility of the work that we publish. This form provides structure for consistency and transparency in reporting. For further information on Nature Portfolio policies, see our Editorial Policies and the Editorial Policy Checklist.

Statistics

For all statistical analyses, confirm that the following items are present in the figure legend, table legend, main text, or Methods section.

n/a	Confirmed	
☐	☒	The exact sample size (*n*) for each experimental group/condition, given as a discrete number and unit of measurement
☐	☒	A statement on whether measurements were taken from distinct samples or whether the same sample was measured repeatedly
☐	☒	The statistical test(s) used AND whether they are one- or two-sided<br>*Only common tests should be described solely by name; describe more complex techniques in the Methods section.*
☐	☒	A description of all covariates tested
☐	☒	A description of any assumptions or corrections, such as tests of normality and adjustment for multiple comparisons
☐	☒	A full description of the statistical parameters including central tendency (e.g. means) or other basic estimates (e.g. regression coefficient) AND variation (e.g. standard deviation) or associated estimates of uncertainty (e.g. confidence intervals)
☐	☒	For null hypothesis testing, the test statistic (e.g. *F*, *t*, *r*) with confidence intervals, effect sizes, degrees of freedom and *P* value noted<br>*Give P values as exact values whenever suitable.*
☒	☐	For Bayesian analysis, information on the choice of priors and Markov chain Monte Carlo settings
☐	☒	For hierarchical and complex designs, identification of the appropriate level for tests and full reporting of outcomes
☐	☒	Estimates of effect sizes (e.g. Cohen's *d*, Pearson's *r*), indicating how they were calculated

Our web collection on statistics for biologists contains articles on many of the points above.

Software and code

Policy information about availability of computer code

Data collection	Vendor supplied MRI console software (Siemens Trio and Siemens Prisma Fit) for acquisition of T1 weighted MPRAGE and resting state EPI sequences.<br><br>Siemens syngo and in house PACS/DICOM servers for raw image archiving and export.<br><br>REDCap (Research Electronic Data Capture) for secure entry and management of demographic, neuropsychological, and clinical data (e.g., CDR SB, MMSE).<br><br>Excel/CSV templates with automated validity checks for on site behavioral data entry.<br><br>No custom software was required for data acquisition; all custom code was used solely in downstream preprocessing and analysis (see “Data analysis” section).
Data analysis	fMRIPrep (version 21.0.0) for initial preprocessing of functional MRI data (e.g., motion correction, slice timing, registration, normalization)<br><br>FSL (version 6.0.5) for subsequent bandpass filtering and confound regression (e.g., fslmaths, fslglm)<br><br>AFNI (version 21.12.19) for post-processing steps (e.g., 3dTshift, 3dBandpass)<br><br>SPM12 (version r7771) for structural MRI segmentation and longitudinal registration

MATLAB (version R2022b) for partial least squares regression, custom scripts implementing dimensionality reduction (PCA), and dynamical systems modeling (eigendecomposition of coupled harmonic oscillator systems)

Python (version 3.10) for ridge regression, cross-validation; primary libraries included scikit-learn and NumPy/pandas

ComBat for harmonizing regional atrophy and functional connectivity data across different scanners

Custom code:
All code for data integration, partial least squares regression, and eigenmode analysiswas written in Python and MATLAB. These scripts are available at https://github.com/jbrown81/structure_function.

For manuscripts utilizing custom algorithms or software that are central to the research but not yet described in published literature, software must be made available to editors and reviewers. We strongly encourage code deposition in a community repository (e.g. GitHub). See the Nature Portfolio guidelines for submitting code & software for further information.

Data

Policy information about availability of data

All manuscripts must include a data availability statement. This statement should provide the following information, where applicable:

- Accession codes, unique identifiers, or web links for publicly available datasets
- A description of any restrictions on data availability
- For clinical datasets or third party data, please ensure that the statement adheres to our policy

Source data are provided with this paper. All data supporting the figures and tables in this study are available as: 1) a MATLAB file named adftd_structure_function_data.mat containing all necessary data variables on Zenodo at https://doi.org/10.5281/zenodo.16783268 with a legend at https://github.com/jbrown81/structure_function/blob/main/adftd_structure_function.m, 2) Text (.txt) files for neuropsychological data at https://github.com/jbrown81/structure_function/tree/main/cognition_data, or 3) NIfTI (.nii) files for activity gradient maps at https://github.com/jbrown81/structure_function/tree/main/gradient_maps.

Raw MRI scans from the UCSF cohort can be obtained from the UCSF Memory and Aging Center under a data use agreement approved by the UCSF Institutional Review Board. The replication dataset analyzed in this study is available through the Alzheimer's Disease Neuroimaging Initiative (ADNI) after registration and compliance with ADNI data use policies (https://adni.loni.usc.edu/).

Research involving human participants, their data, or biological material

Policy information about studies with human participants or human data. See also policy information about sex, gender (identity/presentation), and sexual orientation and race, ethnicity and racism.

Reporting on sex and gender	Biological sex (male/female) was self reported during enrollment. The UCSF cohort comprised 179 females and 142 males; the ADNI replication cohort comprised 277 females and 200 males. Sex was included as a covariate in all statistical models; no sex stratified analyses were pre specified. No gender identity data were analyzed in this study.
Reporting on race, ethnicity, or other socially relevant groupings	Self identified race/ethnicity was recorded at enrollment using UCSF electronic medical record categories. In the discovery cohort, participants identified as 25 Asian, 5 Black/African American, 5 Hispanic/Latinx, 263 White, and 23 "other/unknown." Race/ethnicity was used only for demographic description and not as an analytic variable; therefore no race based statistical comparisons were performed.
Population characteristics	Participants were 221 patients with dementia syndromes (Alzheimer's disease, behavioural variant frontotemporal dementia, corticobasal syndrome, semantic and non fluent variant primary progressive aphasia) and 100 cognitively normal controls (mean age = 66 ± 9 years, range 45–85). Key clinical variables (age, sex, diagnosis, CDR SB, MMSE) are provided in Table 1. Replication analyses used 477 ADNI3 scans from 421 CN and 56 AD dementia participants.
Recruitment	Patients were recruited through the UCSF Memory and Aging Center and met consensus diagnostic criteria at a research clinic visit. Cognitively normal controls were recruited from the UCSF Hillblom Healthy Aging Study. The ADNI dataset was obtained from the public ADNI repository after institutional registration. Participation was voluntary; potential self selection bias (e.g., higher education, interest in research) is consistent with typical academic medical research centers in metropolitan areas.
Ethics oversight	The UCSF Institutional Review Board approved all study procedures. Written informed consent was obtained from all participants or their legally authorized representatives. ADNI data were collected under local IRB approvals at each contributing site and are shared in accordance with ADNI data use policies.

Note that full information on the approval of the study protocol must also be provided in the manuscript.

Field-specific reporting

Please select the one below that is the best fit for your research. If you are not sure, read the appropriate sections before making your selection.

☒ Life sciences ☐ Behavioural & social sciences ☐ Ecological, evolutionary & environmental sciences

For a reference copy of the document with all sections, see nature.com/documents/nr-reporting-summary-flat.pdf

Life sciences study design

All studies must disclose on these points even when the disclosure is negative.

Sample size	No formal power calculation was performed; we analysed all eligible participants who met inclusion criteria. The UCSF cohort comprised 221 patients across five dementia syndromes and 100 cognitively normal controls, numbers that provided stable results with cross validation. An independent ADNI replication dataset (421 cognitively normal and 56 AD dementia participants; 35 imaging sites) was included to verify generalizability.
Data exclusions	Pre specified exclusion criteria were: (i) excessive head motion on fMRI (mean framewise displacement > 0.55 mm; 12.2% of scans), (ii) structural MRI with severe artefact or failed segmentation, (iii) low confidence or unstable clinical diagnosis, and (iv) outlier connectivity patterns identified by data driven PCA QC (20.6% of scans).
Replication	Reproducibility was addressed at three levels:<br><br>1. Internal resampling.<br><br>Split half analysis: 1,000 random, syndrome balanced half splits of the UCSF discovery cohort showed high reliability for structural components S1–S3 (median loading vector correlations: 0.93, 0.88, 0.77; Supplementary Fig. 1).<br><br>Cross?validated ridge regression: Four?fold cross?validation (1,000 trials) yielded significant out?of?sample correlations between atrophy and functional scores for all three components (median r = 0.49, 0.32, 0.39; Supplementary Fig. 3A), and ridge?derived FC weights closely matched PLS weights (median component?wise r ≥ 0.68; Supplementary Fig. 3B).<br><br>2. Independent cohort replication (external dataset).<br><br>We applied the UCSF?derived component loadings to 821 structural/functional scans from the ADNI3 database collected at 35 sites (421 cognitively normal, 56 AD dementia). After ComBat harmonization, structure?function correlations remained significant for components 1–3 (partial r = 0.25, 0.15, 0.08; all P ≤ 0.015; Supplementary Fig. 4).<br><br>The primary functional biomarker (F1) predicted CDR SB in ADNI (variance explained = 33.2%; strongest terms S1, F1, F3, S2; Methods), mirroring discovery cohort results.<br><br>3. Longitudinal consistency.<br><br>In a subset of 53 participants scanned twice (mean interval ≈ 1.1 years), within?subject changes in S1 and F1 correlated with changes in CDR?SB (t ≈ 2.1, P = 0.04) and with each other, indicating temporal stability (Supplementary Fig. 5).<br><br>These internal, external, and longitudinal checks confirm that the structure function components and their behavioural associations are robust and generalizable.
Randomization	This was an observational study; participants were not randomly allocated to groups. Group comparisons controlled for covariates (age, sex, education, scanner type, head motion) in all statistical models.
Blinding	Imaging preprocessing and statistical analyses were automated and script based, minimizing investigator bias; however, investigators were not blinded to diagnostic group during analysis because group labels were required to construct syndrome specific contrasts.

Reporting for specific materials, systems and methods

We require information from authors about some types of materials, experimental systems and methods used in many studies. Here, indicate whether each material, system or method listed is relevant to your study. If you are not sure if a list item applies to your research, read the appropriate section before selecting a response.

Materials & experimental systems

n/a	Involved in the study	
☒	☐	Antibodies
☒	☐	Eukaryotic cell lines
☒	☐	Palaeontology and archaeology
☒	☐	Animals and other organisms
☒	☐	Clinical data
☒	☐	Dual use research of concern
☒	☐	Plants

Methods

n/a	Involved in the study	
☒	☐	ChIP-seq
☒	☐	Flow cytometry
☐	☒	MRI-based neuroimaging

Plants

Seed stocks

Report on the source of all seed stocks or other plant material used. If applicable, state the seed stock centre and catalogue number. If plant specimens were collected from the field, describe the collection location, date and sampling procedures.

Novel plant genotypes

Describe the methods by which all novel plant genotypes were produced. This includes those generated by transgenic approaches, gene editing, chemical/radiation-based mutagenesis and hybridization. For transgenic lines, describe the transformation method, the number of independent lines analyzed and the generation upon which experiments were performed. For gene-edited lines, describe the editor used, the endogenous sequence targeted for editing, the targeting guide RNA sequence (if applicable) and how the editor was applied.

Authentication

Describe any authentication procedures for each seed stock used or novel genotype generated. Describe any experiments used to assess the effect of a mutation and, where applicable, how potential secondary effects (e.g. second site T-DNA insertions, mosiacism, off-target gene editing) were examined.

Magnetic resonance imaging

Experimental design

Design type: Task free resting state design with eyes closed plus structural (T1 weighted) imaging.

Design specifications: Resting state runs lasted 8 min 06 s (240 vols, TR = 2 s, Siemens Trio) or 8 min 05 s (560 vols, TR = 0.85 s, Siemens Prisma multiband ×6).

Behavioral performance measures: No behavioral task was performed during scanning.

Acquisition

Imaging type(s): High resolution structural (T1 weighted MPRAGE) and gradient echo EPI resting state functional MRI.

Field strength: 3T (Siemens Trio and Siemens Prisma Fit).

Sequence & imaging parameters: Subjects received T1-weighted magnetization-prepared rapid gradient echo structural MRI (MPRAGE) scans with similar acquisition parameters on the Trio or Prisma: acquisition time: 8:53; sagittal slice orientation; thickness: 1.0 mm; field of view: 160x240x256 mm; isotropic voxel resolution: 1mm3; TR: 2300 ms; TE: 2.98 ms for Trio, 2.9 ms for Prisma; TI: 900 ms, flip angle: 9°.
Task-free fMRI scans were run using a T2*-weighted echoplanar scan with subjects instructed to remain awake with their eyes closed. The parameters on the Trio were: acquisition time: 8:06; axial orientation with interleaved ordering; field of view: 230x230x129 mm; matrix size: 92x92, effective voxel resolution: 2.5x2.5x3.0 mm; TR: 2000 ms, for a total of 240 volumes; TE: 27 ms. For the Prisma, the fMRI parameters were: acquisition time: 8:05; axial orientation with interleaved multi-slice mode and multiband acceleration=6; field of view: 211x211x145 mm; matrix size: 92x92, effective voxel resolution: 2.2x2.2x2.2 mm; TR: 850 ms, for a total of 560 volumes; TE: 33 ms.

Area of acquisition: Whole brain coverage.

Diffusion MRI: ☐ Used ☒ Not used

Preprocessing

Preprocessing software: fMRIPrep 21.0.0 (incorporating FSL 6.0.5 & AFNI 21.12.19) and SPM12 (r7771) for structural pipelines.

Normalization: All images were non linearly warped to a common template using ANTs within fMRIPrep.

Normalization template: MNI152NLin6Asym (ICBM 2009c).

Noise and artifact removal: 24 parameter motion, white matter and CSF regressors (with derivatives/quadratics) plus 0.008–0.08 Hz band pass filtering and scanner harmonization via ComBat.

Volume censoring: No frames were scrubbed; scans with mean FD > 0.55 mm were excluded.

Statistical modeling & inference

Model type and settings: ROI wise multivariate partial least squares and ridge regression models linked 246 region atrophy vectors to 30,135 edge functional connectivity matrices.

Effect(s) tested: Associations between structural components, functional connectivity components, and clinical severity (CDR SB/MMSE).

Specify type of analysis: ☐ Whole brain ☐ ROI-based ☒ Both

Anatomical location(s) | Regions defined by the 246 node Brainnetome atlas (210 cortical, 36 subcortical).

Statistic type for inference | Component wise Pearson correlations with 1000 iteration split-half cross-validation.

(See Eklund et al. 2016)

Correction | False discovery rate (Benjamini–Hochberg, q = 0.05) across cognitive models and predictors.

Models & analysis

n/a	Involved in the study
☐	☒ Functional and/or effective connectivity
☒	☐ Graph analysis
☐	☒ Multivariate modeling or predictive analysis

Functional and/or effective connectivity | Pearson correlation.

Multivariate modeling and predictive analysis

Multivariate modelling and predictive analysis
Independent variables. Primary structural predictors were 246 element regional atrophy (W score) vectors, harmonized across scanners with ComBat; primary functional predictors were 30,135 Fisher z transformed functional connectivity edges (upper triangle of the 246 × 246 matrix). Age, sex, education, scanner type and mean framewise displacement were included as covariates in all behavioral models.

Feature extraction & dimension reduction.
(i) Partial least squares regression (PLSR; MATLAB plsregress, 5 components, z scored data) linked atrophy and connectivity, yielding subject wise structure and function scores.
(ii) For robustness, ridge regression (sklearn.linear_model.Ridge, α = 1000) was trained on PCA?derived atrophy components and predicted FC scores.
(iii) fMRI time series were projected onto a 6 gradient PCA basis derived from an independent control cohort; eigenmode decomposition of a coupled oscillator model provided gradient amplitude/phase features.

Models.
- PLSR and ridge regression for structure–function mapping.
- Linear discriminant analysis (sklearn.discriminant_analysis) to classify "typical" syndrome patterns.
- Generalized additive models (mgcv in R) to predict 24 neuropsychological scores from S1–S3 and F1–F3 while adjusting covariates.
- Mixed effects GAMs for longitudinal change and for external ADNI replication (random intercepts for subject/site).

Training & evaluation.
- Four fold stratified cross validation (1,000 repeats) for ridge models; performance reported as out of sample Pearson r between predicted and observed FC scores.
- Split half resampling (1,000 iterations) for PLS component stability (median region loading correlation).
- External replication: partial correlations between atrophy and FC scores in 821 ADNI scans.
- Permutation testing (10,000 label shuffles) for all structure–function correlations; significance set at FDR corrected $q < 0.05$.
- Behavioural models evaluated by adjusted R^2 and F statistics (predictor specific).
- Longitudinal within subject models assessed with t values for change terms and marginal R^2.
